# Supplementary material for: Novel Insights into the Biochemical Mechanism of CK1ε and its Functional Interplay with DDX3X
Source: Int J Mol Sci. 2020 Sep 3;21(17):6449. doi: 10.3390/ijms21176449 (PMC7503845; doi:10.3390/ijms21176449)
Supplement: Supplementary file 1 [file ijms-21-06449-s001.pdf]

Novel insights into the biochemical mechanism of CK1ε and its functional interplay with DDX3X

Bartolo Bono[1] , Giulia Franco[1], Valentina Riva[1], Anna Garbelli[1] and Giovanni Maga[1,*]

[1] Institute of Molecular Genetics IGM-CNR "Luigi Luca Cavalli-Sforza", via Abbiategrasso 207, 27100 Pavia (Italy)
* Corresponding author: Dr. Giovanni Maga; E-mail: giovanni.maga@igm.cnr.it; Tel: +390382546322

Supplementary Information

Supplementary Figures 1A-D: original images for Blots and Gels Requirements;

Supplementary Figures 1E-F Effect of the presence of DDX3X on Dvl2 phosphorylation by CK1ε

Supplementary Figure 1A: Original images for figure 6 A

Autoradiography

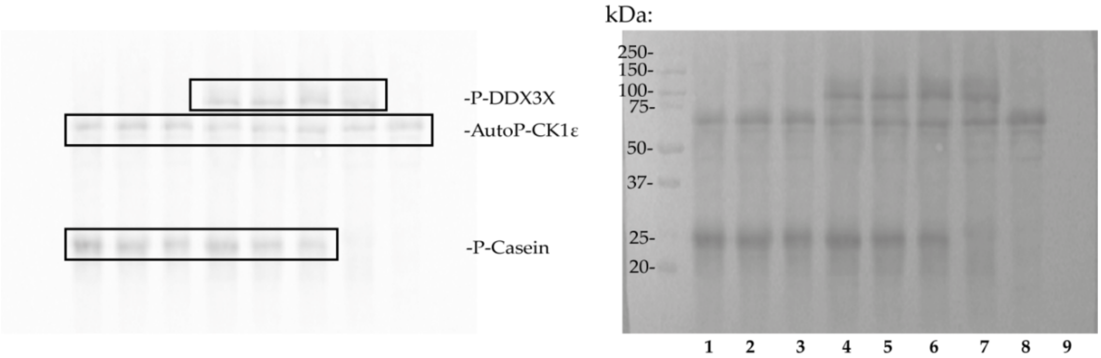

Uncropped and unadjusted western blotting images

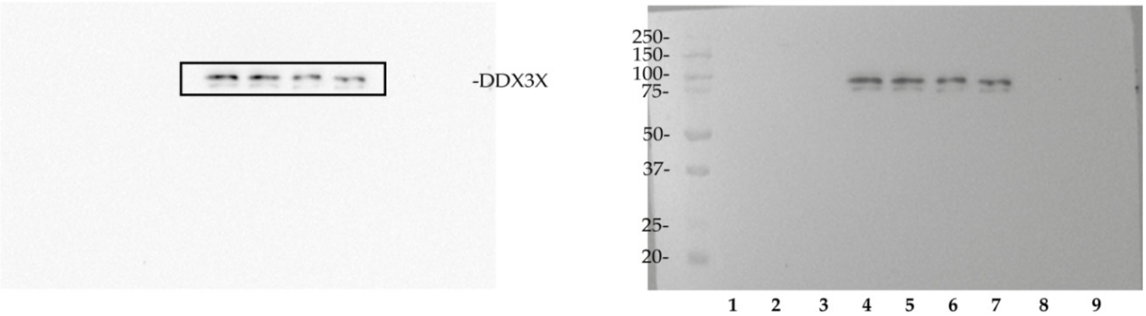

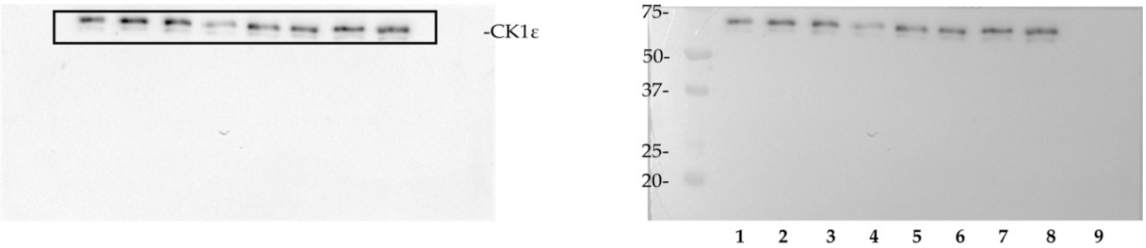

Supplementary Figure 1B: Original images for Figure 6 C

Autoradiography

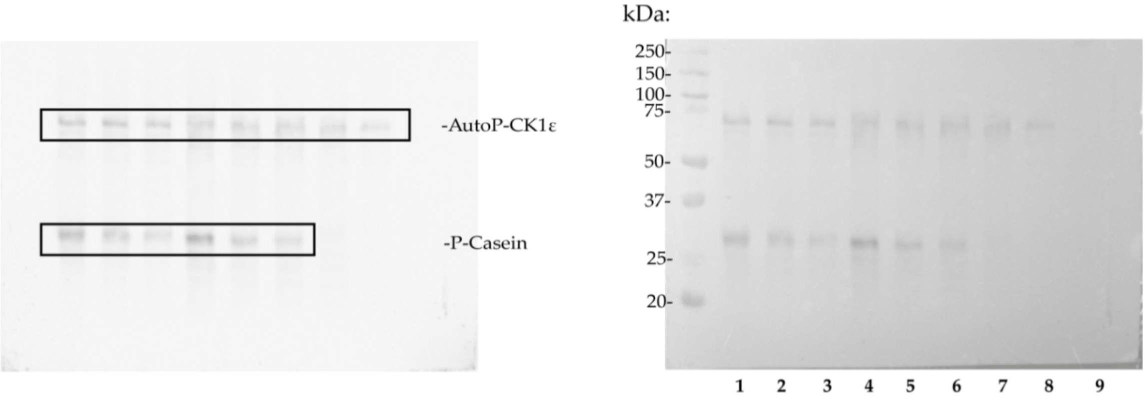

Uncropped and unadjusted western blotting images

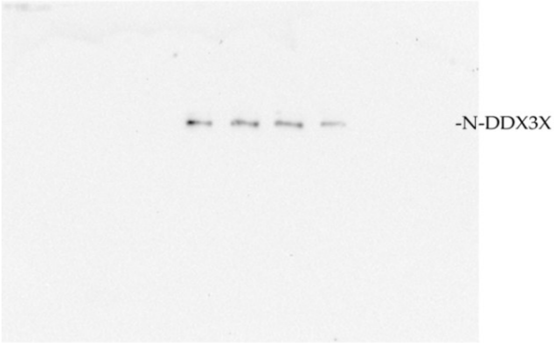

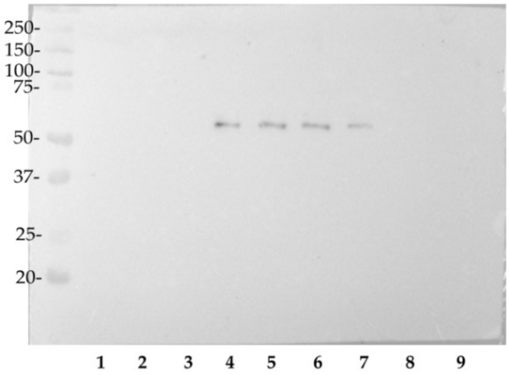

Supplementary Figure 1 C-D: Original images for Supplementary Figure 1 E-F

Autoradiography

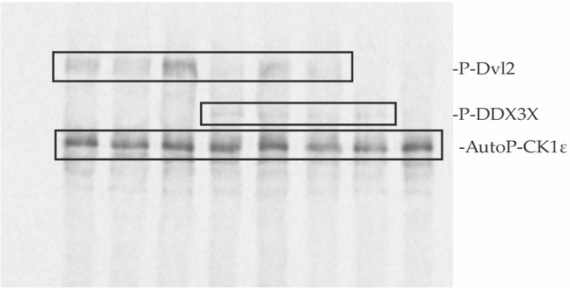

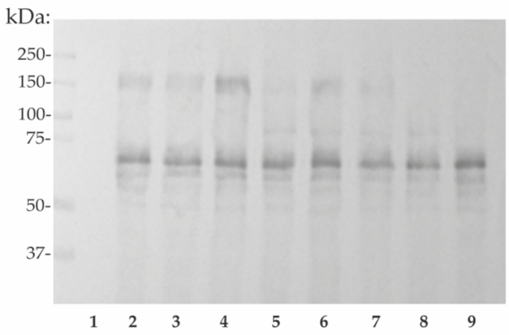

Western Blotting

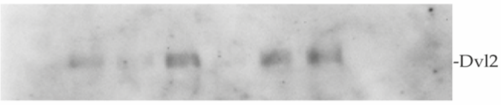

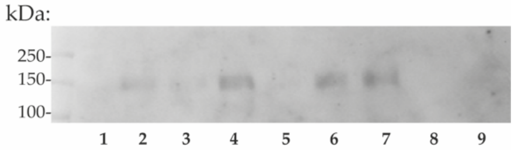

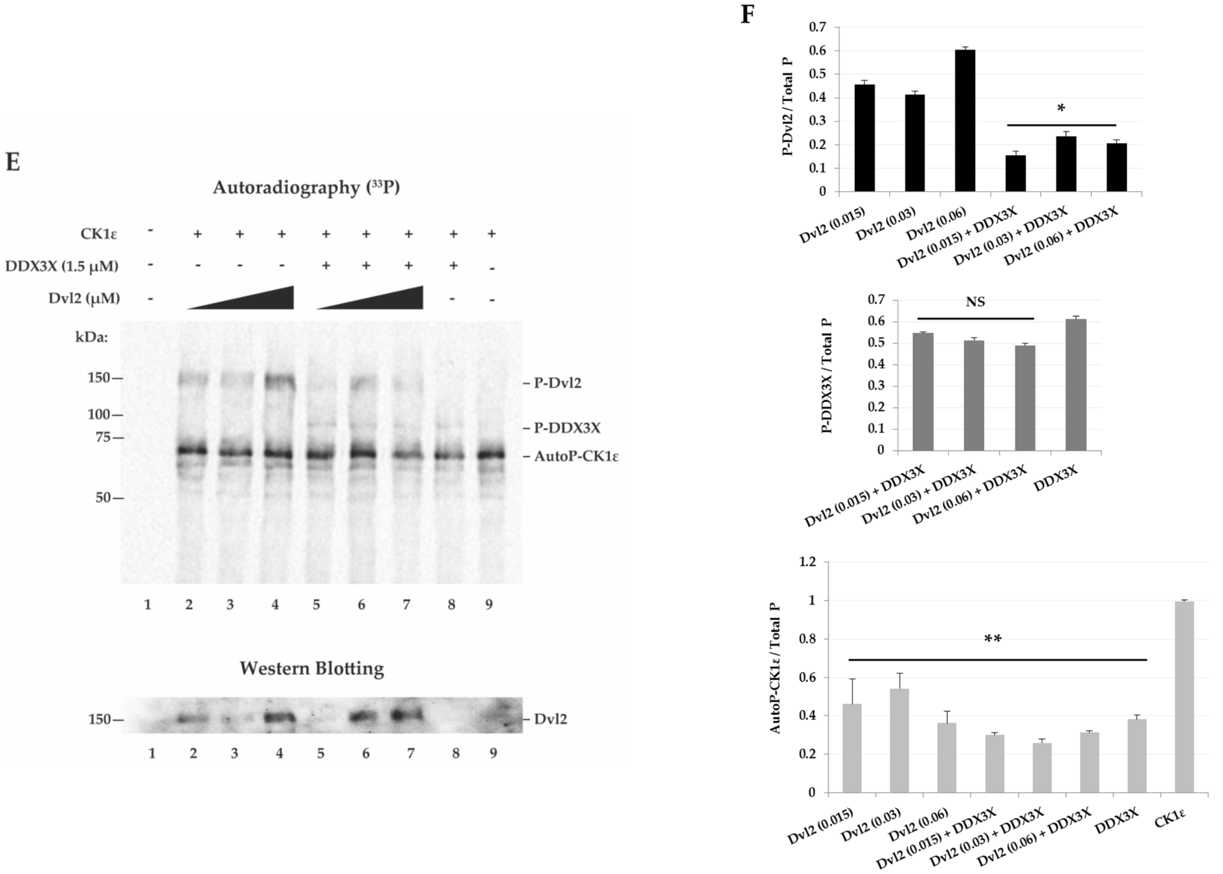

Supp. Figure 1 E-F. Effect of the presence of DDX3X on Dvl2 phosphorylation by CK1ε. (**E**) DDX3X, CK1ε and Dvl2 γ-^{33}P incorporation visualized by autoradiography on nitrocellulose membrane (upper panel). Western blot analysis of Dvl2 protein of the same membrane (lower panel). (**F**) Quantification of autoradiographic γ-^{33}P signal (panel E). Bars indicate mean ± SD. * and ** indicate *p*-value < 0.05 and < 0.01 respectively. NS: not significant.
